# Trouble with autonomy in behavioral insurance

**DOI:** 10.1111/1468-4446.12960

**Published:** 2022-06-21

**Authors:** Maiju Tanninen, Turo‐Kimmo Lehtonen, Minna Ruckenstein

**Affiliations:** ^1^ Centre for Consumer Society Research University of Helsinki Helsinki Finland; ^2^ Tampere University Tampere Finland

**Keywords:** autonomy, datafication, ethico‐political deliberation, insurance, self‐tracking, values

## Abstract

In this article, we study how people define, negotiate, and perform autonomy in relation to digital technologies, specifically in connection with behavioral insurance policies that involve forms of data tracking and health services. The article builds on focus group discussions, which we treat as a dynamic site of ethico‐political deliberation to test ideas, talk about boundaries of acceptable control, and envision future scenarios. The ethico‐political deliberations assess the legitimacy and usability of new behavioral tools. Concern over the nature and limits of autonomy is activated when people discuss how wellbeing‐related decisions are delegated to algorithmically controlled systems. We argue for appreciating autonomy as a relational and ambiguous notion that is sensed and enacted in collaborations with devices in the form of distributed autonomy. Moreover, as reflected by the experiences of the insured, “autonomy” cannot be analyzed solely in the form transmitted by the liberal tradition; that is, as a clear‐cut entity that can simply be “had”, “exerted”, or “controlled”. Consequently, research, ethical considerations, and governance initiatives should pay attention to how values are “done” in the affect‐laden technologically mediated relations and practices.

## 1 INTRODUCTION

Current socio‐technical developments characterized by the proliferation of digital infrastructures and data‐intensive automated services have created a situation in which commonly shared values ranging from solidarity and autonomy to trust and equality are viewed as under threat (Prainsack & Van Hoyweghen, 2020; Sharon, 2018). Not coincidentally, debates around privacy, fairness, transparency, and accountability are multiplying. These in turn promote legal, regulatory, and ethical frameworks and new governance initiatives (Marelli et al., 2020). The recent proposal for harmonized rules on artificial intelligence in the EU (European Commission, 2021) is only one regulatory attempt to exploit the socio‐economic benefits and mitigate the harms related to algorithmic systems.

The latest governance initiatives are ambitious in their goals but leave much to be desired from the perspective of the social sciences and humanities. They typically employ limited conceptions of value that ignore decades of research that would enable an exploration and reimagination of the different facets of values invested in current algorithmic systems (Graeber, 2001; Sykes, 2018). Rather than retaining the flexibility of ethical considerations to react to emerging practices in data‐intensive automated technologies, ethics can be reduced to codes of conduct (Rességuier & Rodrigues, 2020). For instance, predetermined and narrow notions of values are located within algorithmic operations (fairness as a statistical property of models), ignoring how they are situationally understood and practised in the larger contexts in which algorithmic systems are embedded (Lanzeni & Pink, 2021).

The present socio‐technical moment calls for social scientists to intervene in the ongoing debate by drawing on anthropology, sociology, and the interdisciplinary field of valuation studies to examine how values are deployed in situated practices rather than in the abstract. In a practice‐based understanding, values are located in the complex ways that technical arrangements mediate human agencies and vice versa (Dussauge et al., 2015; Fourcade, 2011; Helgesson & Muniesa, 2013). In the present study, we exemplify what this broader notion of values adds to the ongoing debate on algorithmic systems by studying behavioral life insurance products in Finland. We demonstrate how one value in particular—autonomy—becomes mobilized in relation to such insurance products, as they push against notions of what insurance should do. We “stay with the trouble”, as the much used trope by Donna Haraway (2016) goes, to appreciate the nature of autonomy as an emergent and relational value that responds to the shifting circumstances of people's lives.

After a long historical evolution, autonomy as a concept has come to dominate the contemporary discourse on liberty, freedom of will, and self‐determination (Honneth, 2015; MacPherson, 1962; Sulkunen, 2009; Taylor, 1989). Instead of regarding “autonomy” as something universal and thus immutable, we suggest an alternative approach in which autonomous conduct “varies conceptually and materially over time” and is shaped by the prevalent “cultural equipment” with which it is performed (du Gay, 2005, pp. 395–396). Consequently, the idea of autonomy also provides an invaluable lens to examine how people situate themselves in the emerging socio‐technological landscape through their personal experiences.

In the context of behavioral insurance, that is, insurance products that include forms of data tracking and behavioral intervention, autonomy often operates as an appeal to self‐determination and is treated in a manner akin to what Michel Foucault (1978) defined as the juridical notion of power. The juridical understanding of autonomy is also a built‐in feature of conventional insurance (Ewald, 1986) and resonates with the experiences people have with behavioral insurance. Thus understood, “autonomy” is an entity that a person can “have” or “exert” and that others can “control”. At the same time, the juridical understanding of individual autonomy is insufficient for examining how autonomy emerges or is threatened in insurance‐related practices. For this, we need a relational understanding of autonomy that stems from practice‐based understandings of values and feminist ethics (Mackenzie, 2008; Mackenzie & Stoljar, 2000; Westlund, 2009). This broader notion of autonomy helps answer the question of what exactly algorithmic technologies—in this case novel forms of insurance—are doing to us and what we are doing to them. Here, we seek to understand how socio‐technical change “creates new ways for people to be”, in Ian Hacking's words (1986, p. 161). Importantly, the novel forms of becoming with insurance are intimately tied to “the space of possibilities” (Hacking, 1986, p. 165) where autonomy is shaped by algorithmic relations. These relations are defined by intensive human‐machine interactions that make holding on to clearly bounded juridical notions of self‐determination difficult, if not impossible (Cevolini & Esposito, 2020; Hayles, 2017; McFall & Moor, 2018; Sharon, 2017).

Below, we examine how experiences with behavior‐based insurance products inform us about defining, negotiating, and “doing” autonomy. We argue that in addition to appreciating the work that the juridical notion of autonomy does in guarding the limits of self‐determination, we need to examine relational understandings of autonomy in the specific context of socio‐technical arrangements. The first approach, which aligns with current regulatory perspectives, delineates a field where autonomy materializes in a fairly orderly manner, as people protect and nurture their free will and make informed decisions about using technology. By contrast, the second approach forces us to consider the situational and contextual aspects of autonomy, which are more difficult to perceive, let alone manage. Together, the two approaches demonstrate how people try to get a grip on the current socio‐technical landscape, one technological relation at a time. Their discrepancies demonstrate the limitations of current regulatory and ethical approaches that ignore the emergent, relational, and distributed aspects of autonomy. Thus, we offer a way forward in the current debate by identifying which aspects of autonomy can be clearly bounded and which need a more reflexive approach.

## 2 BEHAVIORAL INSURANCE AS AN EXEMPLARY SITE

While the industry celebrates the potential of new technologies to enhance and strengthen individual autonomy as service providers seek to gently guide people to make better decisions, others doubt such empowering effects. For instance, hypernudging—the use of data‐driven personalized choice architectures that strive to affect consumers' behavior—has been criticized as a form of manipulation (Yeung, 2017) and an invasion of people's decisional privacy (Lanzing, 2019; Zuboff, 2019). What is notable in light of our inquiry is that both enthusiastic and critical perspectives appeal to the value of individual autonomy (Sharon, 2015, 2017). As we show below, the relevant understanding of autonomy that emerges in practice calls for a broader approach; we need to circumvent the tendency to draw conclusions about algorithmic technologies based on dichotomous responses. To do so, we examine the value of autonomy by way of two behavior‐based life insurance policies introduced to the Finnish market in the late 2010s by insurers we call Company X and Company Z.

The studied insurance policies combine regular life insurance with digital services produced by partnering with health analytics companies and include forms of self‐tracking conducted with smartphones, activity wristbands, or smartwatches. In practice, the Finnish insurers gather the data generated by the different devices with the help of the analytics companies, which “purify” the information of excessive details and glitches and select certain variables for the insurers' use; through this arrangement, the insurers seek to collect enough data to fulfill the policies' purposes while still complying with insurance regulations.

The insurance companies' incentives to promote behavior‐based policies are threefold: first, the information collected could be used to fine‐tune risk management; second, the policies function as a marketing tool to improve customer relationships and retention; and finally, if the new devices help users engage in healthier practices, the number and total amount of claims can be expected to decrease (Jeanningros, 2020; McFall 2019; Tanninen et al., 2021). Because of legal, market, and technological constraints, the data produced by means of tracking activities are not deeply integrated into the insurance mechanisms of risk pooling, underwriting, and pricing. As Jeanningros and McFall (2020, p. 2) note, “self‐tracking data is of marginal importance” in the health and life insurance sector. Still, the data are used in the bonus structure of Company Z's policy: if customers reach a certain activity level, they are guaranteed a modest increase in their insurance coverage. Company X, meanwhile, was at the time of us conducting the research planning a reward structure for its policy but relied more than Company Z on the “hypernudges” embedded in the health services to which the policy provides access.

The Finnish insurance products represent a wider business trend characterized by rapid change and fervent experimentation: especially in the fields of car, health, and life insurance, companies invent and test new forms of business that employ the presumed advantages of digital mediation in consumer relations (Barry & Charpentier, 2020; Cevolini & Esposito, 2020; Meyers & Van Hoyweghen, 2020). Yet, the two policies studied here are not so much instances of something widespread in a new insurance field; rather, they are experimental sites where novel developments are tested. The successful merger of established financial rationality and new digital infrastructures, including self‐tracking practices, cannot be taken for granted: failure *is* an option. Rather than anticipating an inevitable expansion of behavior‐based insurance, we are interested in the way it probes the limits of autonomy and raises questions about the kind of society we want to inhabit and who we want to become in the process.

In exchange for reporting customers' experiences and perceptions to the companies, we were able to form a connection to policyholders, a group that is otherwise difficult to reach. The data for the present research were gathered by Maiju Tanninen (MT), who conducted a total of 11 focus group discussions in autumn 2017 and spring 2019. As legal restrictions limited information sharing, the insurance companies recruited study participants. If the companies had purposively chosen them, this could have negatively affected the validity of our results. As recruitment proved to be challenging, however, the selection process turned out to be quite random.

The participants included individuals with behavior‐based policies and those with regular life insurance policies. Additionally, MT facilitated three focus groups with people who did not have life insurance policies but were perceived as potential customers by the market research panels through which they were recruited. Each focus group had between two and eight participants. Altogether, 46 people took part (24 women and 22 men), ranging in age from their late twenties to their sixties. The discussions spanned from 45 to 90 minutes and were recorded and transcribed. In the analysis, participant names were pseudonymized.

## 3 FOCUS GROUPS AS SITES OF ETHICO‐POLITICAL DELIBERATION

Focus group discussions have the advantage of facilitating interaction between participants and inviting a greater variety of communication than individual interviews (Kitzinger, 1995). Group discussions enable people to compare and nuance their viewpoints; the multi‐vocal accounts that result are not only richer but also more speculative than standard interviews. Still, the interactional nature of focus group discussions is often disregarded, with data treated as a set of multiple individual interviews: researchers might choose extracts of what a given person has said and ignore the situation in which those utterances were made. This focus on single statements may stem from the market research history of focus group discussions; in that setting, the method's main purpose was to generate tradable and “authentic” individual opinions (Lezaun, 2007). Robert Merton, a focus group pioneer, however, envisioned the method as suitable for examining “every sphere of human behavior and experience”, not just the interests of market research. He emphasized how interaction among participants “served to elicit the elaboration of responses” and introduced “new leads stimulated by others” (1987, pp. 555, 562). In fact, researchers have increasingly highlighted the need to consider the interactional context of focus group discussions and the conversational dynamics at play; it is difficult to understand *what* people are saying if one ignores *how* they are saying it (Grønkjær et al., 2011; Halkier, 2010).

Our analysis draws on these insights, as we are particularly interested in the collective action and imagining that occurred in the focus groups. In practical terms, we treat the discussions as *ethico‐political deliberation:* participants tested their ideas, discussed the boundaries of (real and imagined) mechanical control, and envisioned future scenarios. We have allowed the interactional dynamics to guide our exploration, focusing on the moments when conversation sped up and began to take on a life of its own. This typically occurred when participants jointly speculated on the future of data‐driven technologies, often enthusiastically and humorously, building on one another's utterances. We have treated intensified interactional pace as an indication that crucial issues were at stake; the fact that people so often elaborated on others' remarks suggests that they contemplated a wide repertoire of ideas, meanings, experiences, and fears related to this type of technological development.

Thus, we see the focus group as a particularly useful setting for examining an emerging technology with unsettled ethical and political aspects; it works as a “provocative containment” that both produces and displays social reality, surfacing “something not readily available” while handling it in a “clearly demarcated place” (Lezaun et al., 2013, pp. 279–280). In our case, the moments of excited discussion appeared to revolve around tensions between free will and the controlling measures that the new technologies could support. Importantly, it was not our original aim to generate discussions around autonomy; rather, complex ethico‐political deliberations surfaced organically in the conversations, emphasizing that autonomy was viewed as under threat from the intrusive nature of new forms of insurance. In everyday speech, people rarely use the term “autonomy”, as it is a taken‐for‐granted value that organizes talk rather than something that needs to be defined. What we recognize as “autonomy” emerged and became explicit in the focus group discussions when people spoke of self‐determination, free will, doing something voluntarily, being in control, or feeling that they are under surveillance. For our analysis, we refer to a discourse‐centered approach, arguing that the discourse circulating in a social entity constructs the world in which the entity—in this case autonomy—situates itself (Urban, 1991). It is typical of all values to become invigorated and observable when endangered, and the talk about autonomy vividly makes this point.

Below, we stay with the trouble of autonomy and trace the tensions, negotiations, and practices that constitute self‐determination in the case of behavioral insurance. This line of inquiry offers possibilities for richer assessments than, for instance, the critical data studies approach (Tanninen, 2020). As we are not constrained by an essentialist understanding of individual autonomy, the focus of the critique shifts from the question of whether a particular technology supports or hinders individual autonomy to the question of whether this way of doing autonomy is even desirable. Our approach enables more appropriate ethical considerations than abstract ethical principles: instead of simply branding an entire technology as corrupt and thus perhaps overriding users' viewpoints, a more flexible approach can demonstrate which practices are subject to doubt, ambivalence, and fear and thus why they are worth criticizing.

In the sections below, we first examine *how people discuss* the limits for acceptable manipulation in the context of behavioral insurance. The analysis reveals two contrasting yet simultaneous desires: people “rationally” emphasize their individual autonomy even as they humorously imagine cases of extreme control and express their willingness to distribute some of their decisional power to external forces. The second form in which autonomy comes into being in our analysis focuses on the practical negotiations concerning how living with insurance‐related technologies should be arranged; here, we examine *what takes place in these relations* and how autonomy is sensed and enacted in collaboration with devices. We discuss cases where tracking technologies help people achieve their goals and others where the alignment between consumers and policy objectives is ruptured; that is, the moments when policy features previously experienced as useful become intrusive and annoying.

## 4 THE IMPORTANCE OF CHOICE

In general, the focus group participants did not treat the policies' data tracking features as too invasive or an intrinsic threat to self‐determination. Instead, they reckoned that activity tracking and the health services included in the policies could positively impact their wellbeing. At the same time, the new data relationships created by the insurance schemes did raise uncertainty about the limits of control. In the policies of both Company X and Company Y, the “smart” features are framed as additional services whose use is voluntary. The self‐chosen nature of monitoring appears to be key to its acceptability. Maria and Anne, both Company X customers, agreed that they would not opt for a behavioral insurance policy “if you were obliged to give [your data], wear the [activity] wristband and be monitored”, as Maria put it. For her, existing behavioral policies “feel much nicer” because “there is the opportunity to say no”. Anne added that the ability to choose allows “you to retain your self‐determination” in the insurance relationship. For both women, the ability to choose thus ensured that the data tracking features would not become overly controlling.

In addition to overall acceptability, consumer choice was regarded as ensuring the fairness of the insurance policies' existing and potential reward structures. Rewarding people based on their activity levels seemed reasonable if policies are voluntary (rather than part of social security) and support only positive incentives (instead of punishing people). Samuel, a Company Z customer, thought that it was “quite logical” and “fair” for a private life insurance scheme to financially incentivize its customers, as purchasing that policy is a “voluntary decision”. However, fellow Company Z customers Henri and Camilla challenged Samuel's opinion of the policies' fairness. Henri worried that if data tracking were to become a mandatory element of services, people “won't be able to choose anymore”. For Camilla, the policies already seemed “a little bit unfair” toward chronically ill people, who have a limited ability to move—and thus simply cannot choose to strive for the policy goals. By the end of this interactional sequence, Samuel affirmed the others' concerns. Although he first employed an understanding of deservingness that aligns with the idea of “actuarial fairness”—the notion in the insurance industry that all should pay or be rewarded according to their level of risk—he later agreed with the others, thus underlining the deliberative nature of the focus group context.

Even as the participants emphasized the importance of choice, they also highlighted their ability to assess the limits of these new data relationships, especially their relationships with individual data tracking devices. These devices' basic functionalities imply that they enter the users' intimate space and “nudge” them into alternative behaviors; thus, the question of how receptive and compliant a user is to the devices' output becomes highly relevant to the device–user relationship. Oliver, a Company X customer, underscored that he had not “enslaved himself” to activity tracking—contrary to his colleagues whose need to fulfill daily activity goals no longer sounded “that healthy” to him. Thus, unlike his co‐workers, Oliver framed himself as capable of managing his relationship with the algorithmic tools. Similar stories recurred throughout the discussions. The participants talked about family members, friends, and acquaintances as incapable of setting healthy boundaries for how the devices control them. By contrast, they perceived themselves as able to maintain a balance between the devices' feedback and their own free will. This is a common phenomenon: people routinely evaluate themselves as more capable than others of controlling their behavior (Dogruel et al., 2020).

In addition to discussing individual tracking devices, focus group participants emphasized their self‐sufficiency in relation to the insurance companies' aim to create more intimate and proactive customer relationships through these behavioral policies (Tanninen et al., 2021). For Samuel, the “traditional deal”—where insurance companies simply cover costs when something unexpected happens—was “perfectly fine”, and he did not want “the insurance company's helping hand”. Henri, a fellow Company Z customer, joined the conversation, declaring that he could “manage [his life] quite well”; hence, insurance companies' intensive involvement was not needed. Still, both Samuel and Henri consider the insurance infrastructure an important form of security; they are not so self‐reliant as to opt out of insurance altogether.

These examples suggest that the focus group participants underscored the importance of self‐determination, the ability to choose, and individual autonomy in general; these values emerged in the discussions as crucial for self‐understanding and collective sense‐making. People tended to perceive themselves as freely choosing individuals, capable of managing their lives and determining the limits of their data relationships. Aligning with the logic of choice, as elaborated by Annemarie Mol (2008), “autonomy” appears here as a clear‐cut entity; the individual carefully weighs and balances the consequences of choices made. Although submitting themselves to nudges and having distributed some of their decisional power to external devices, the insurance customers presented themselves as unwilling to submit to forms of control.

## 5 THE DESIRE TO BE CONTROLLED

Alongside an emphasis on individual choice, the participants collectively imagined scenarios in which technologies would force them to manage themselves better. These darkly humorous visions of coercive power present a different rhetoric for dealing with the trouble of autonomy: they enable people to discuss the difficulty of managing their health. For instance, Patrik, who did not have a behavioral insurance policy, imagined a controlling mechanism very similar to the ones that existing self‐tracking devices use, such as a reminder to move after a given period of immobility. However, instead of using typical cues like vibrations or buzzes and relying on their affective impact (such as guilt for not reaching the activity goal), in Patrik's vision a device would either “electrocute the wrist” or have “a spike that goes under the skin”; “the longer you sit, the deeper the spike would go”. Similar tongue‐in‐cheek ideas of extreme control were introduced by many participants, who enthusiastically pictured penalizing tools that would prevent them from engaging in unhealthy habits. These visions, of course, do not prove or even seriously suggest that people would welcome such measures. Instead, they were playfully testing the limits of acceptable control while talking about the difficulty of self‐management with intensified requirements of governing their own health.

Some participants, such as Helena, who was not a behavioral policy customer, imagined all‐encompassing systems that would carry the full burden of self‐governance. She envisaged technologies completely overseeing the management of her wellbeing, such as a tool that would help control her weight by communicating how her eating relates to her exercise habits and calories burned. Thus, instead of merely receiving information about her activity, she would prefer easily understood and actionable advice which she could passively follow. Here, the technological imaginary resonates with the desire to rely on digital technologies to alleviate demands on one's time and to offer motherly guidance and care (Schüll, 2018). Devices are regarded as taking over the customer's will, and the burden of self‐management is distributed to external forces.

The ongoing ethico‐political deliberation became tangible in the discussions in relation to the desire to retain a sense of decisional power. When discussing in a humorous tone visions of extreme mechanical control, participants reiterated the idea that submitting to controlling elements must be voluntary, with opting out always an alternative. This is exemplified by a lively conversation between customers of Company X:

Laura: You would get a small electric shock from the wristband if you went to a fast‐food restaurant.

Daniel: Yeah, “don't touch that bun!”

Jenni: It would be quite nice if you could decide on the control yourself. It would count your calories and when you reached the limit, it would give you terrible shocks; you would only be allowed to drink water.

Laura: Yeah, either you would go for a walk or…

Jenni: As long as you are the one controlling it. If the device starts to control your life, it feels like “hmm, who is the one deciding?” If artificial intelligence takes total control, it could have mistaken ideas about your life and not take everything into consideration.

While the participants here imagine controlling mechanisms, Jenni questioned the ability of datafied tools to “see” people properly, anticipating that these devices would not be capable of considering important aspects of human life. The exaggerated visions show that the trouble with autonomy in behavioral insurance is not solved by setting definitive limits for controlling measures. Instead, two contrasting desires are at stake. On one hand, focus group members emphasized their individual autonomy, insisting that they want to determine their own actions freely, regardless of insurance companies and any self‐management tools on offer. On the other, they used visions of extreme mechanical control to express the difficulty of self‐management and their willingness to accept outside help. The friction between these two desires remains unsettled: the extreme visions playfully poke and test the limits of acceptable control and disturb the straightforward story of individual autonomy.

In the next two sections, we delve more deeply into the intricacies of autonomy by analyzing the practical negotiations regarding arrangements for the relationships between policyholders and insurance technologies. Even in the speculative discussions and elaborations presented above, people struggled to set strict limits on what should remain under an individual's control and what the devices should be allowed to manage. We see here an active balancing of the right “mix between active and passive” (Gomart & Hennion, 1999). By focusing on everyday experiences with the devices, we can further demonstrate that questions of care and control and related activity and passivity comprise a messy, highly contested area that is subject to ongoing, situated negotiations.

## 6 DOING AUTONOMY WITH TECHNOLOGIES

We follow the experiences of two Company X customers to demonstrate how tracking technologies can be understood as helping users achieve their goals. They first wore the activity wristband offered by the insurance policy but then upgraded to more sophisticated devices: Anton uses a smartwatch to track both activity and sleep, while Leo measures his daily activity and running practice with a smartwatch and a separate heart rate monitor. Leo's device is still attached to the insurance policy, but Anton no longer shares his data because his new device fails to communicate with the company's platform. Previously, though, he actively used the insurance‐provided tools.

The men discussed the devices' ability to remind them of wellbeing‐related tasks: getting enough sleep and ensuring daily mobility. Anton described how the smartwatch pushes him “not to spend the whole evening watching TV or reading”; it helps him keep in mind that he has to reach “the sleeping goal”. Here, Anton was not referring to the device's specific nudging features; instead, simply knowing that not sleeping enough would be registered by the smartwatch appears to help him go to bed on time. The more proactive nudging elements were discussed by Anton and Leo in relation to daily activity. Both found the smartwatches' alerts to take breaks from sitting and immobility useful; for instance, Leo described how being reminded in his “static job” is “a good wake‐up call” that urges him to move when the tracker “beeps after an hour”.

When the nudging elements align successfully with the will to pay more attention to immobility, users of controlling gadgets can be relieved of the duty to recall the need to move. This kind of alignment is crucial for well‐adjusted engagement with devices, as it provides tools for “self‐induced nudging into self‐prioritized activities” (Pols et al., 2019, p. 101). Furthermore, the devices can enable new norms and justifications for doing wellbeing: how taking breaks from sitting is a healthy decision.

The nudging elements, however, do not act through a straightforward stimulus–response mechanism, even if they are in principle designed that way. Instead, a great deal of functionality is attributed to their affective impacts, as was evident when Anton and Leo discussed how they feel about tracking. Leo did not acknowledge a guilty conscience for not reaching his activity goals but did say that a low score “motivates [him] to go for a run or a walk” to raise the activity bar “at least a little bit”. For his part, Anton has “set the daily activity goal on purpose to a high level” that he will not reach if he acts in his “usual way”. Setting a high bar effectively means seeking an encounter with an unpleasant feeling, as if its possible appearance is what gets Anton up and moving. Yet, reaching the demanding goal will also be more satisfying, giving Anton a heightened sense of achievement. Anton's case points to self‐nudging with negative (and positive) affects at play. Without unpleasantness, he might not do the extra exercise that eventually brings him joy.

These experiences highlight the fine line between autonomous decisions and the devices' control. For instance, Anton's decision to “self‐nudge” reveals a messy skein of relations. The activity goal that he has chosen was probably a pre‐selected option in his tracking device. In addition, the insurance policies' wellbeing apps have their own goals, which are determined by the companies that handle the data. Finally, the insurance companies fortify the established targets; this is especially true of Company Z, which rewards its customers based on “activity level”. Similar dynamics are at stake with the supervision of the activity targets. The device does the monitoring for the user; it is an externalized gaze, although in the end it is still the people who watch themselves and act (or do not act) on their own data. Externalizing the gaze, however, can help (or hinder) attaining personal goals. As a participant in another focus group put it: “It creates a feeling that you are constantly under a watchful eye. Some are motivated by it; some just get more anxious and depressed”. Finally, the insurance companies participate in the (experienced) surveillance: the firms collect data and, perhaps more importantly, the customers imagine how insurers are—or could be—monitoring them.

Many participants, however, made no connection between their everyday tracking activities and the insurance infrastructure, even in cases where the monitoring tools were provided by the company as part of a policy. The data gathering related to self‐tracking operates silently and in the background, as it is embedded in the larger‐scale digital infrastructure, arranging relations between individuals, devices, and health analytics platforms (Star & Ruhleder, 1996). At the same time as the digital infrastructure maintains these relationships, it efficiently conceals them, with the result that focus group discussions rarely addressed the actual insurance policies. Pauli, a Company Z customer, was an exception in this regard; he explicitly referred to his insurance policy when discussing his tracking activities. Asked by another participant whether it is “a depressing experience to look at [the data]”, Pauli responded that “it is pretty easy to reach the points for the first level of [Company Z's] policy” just by walking to work and back home. What is at stake for Pauli is not so much his personal health but the activity level that makes him eligible for the insurance bonus. He does not actively strive to reach the level; it more or less happens without his trying and is thus a nice fringe benefit. Clearly, in Pauli's case the externalization of control does not evoke intense affects and, thus, Pauli is an example of a successful alignment of customer and policy goals: he reaps the benefits from the policy, and the company has a customer with healthier habits.

These examples show the different ways in which users, data tracking devices, and insurance policies become intertwined: within this complex web, it is not clear who sets the goals and does the monitoring. What is important for this kind of coexistence to work is the feeling that the customer is benefitting from the policy and thus the devices' output: the technologies support achieving health—or insurance—goals. This way, the technologies actively participate in doing a user's sense of self‐determination.

## 7 INTRUSIVE, ANNOYING, AND WEAK CONTROL

Instead of helping people reach wellbeing‐related goals, the insurance‐provided self‐tracking technologies and their nudging elements—blinking lights, buzzes, beeps, and so on—can become a source of disturbance and irritation. The devices do not readily recognize what is happening in customers' lives. Our participants described the tracking technologies' deficiencies in measuring activity: the devices do not register cycling or walks when pushing a stroller, but they do record knitting as physical activity. These inaccuracies were often a laughing matter in group discussions, but they were also a source of genuine frustration, especially when poor device performance prevented policyholders from reaching the activity goals that trigger an insurance bonus.

In addition to failing to recognize physical activity, the devices disregard another important aspect of wellbeing; namely, the need for relaxation (except for sleep). Camilla and Henri, a married couple with small children, discussed how Camilla's employer‐provided activity wristband ruined their evenings with its repeated interruptions:

Henri: When you had the activity wristband, it was a bit annoying when it started to blink and push that “it's time to walk now” [laughter].

Camilla: Yeah, in the evening when we're watching Netflix and…

Henri: Nice and relaxed, when the kids have gone to bed…

Camilla: And then I'm like “okay, maybe I'll take it off now; maybe I deserve to lie on the couch for a couple of hours”.

The irritation that Camilla and Henri reported highlights the device's inability to align its rhythm and suggestions with everyday sociality. It tries to force its internal logic on people's lives and thus fails to recognize the complexity and contingency of human experience and what really matters at a particular point in time: the tracker ruins the pleasurable moment of unwinding by causing negative feelings of annoyance and guilt. That such feelings arise points to the fact that in these moments the mechanical intervention has gone too far: the nudges and suggestions feel intrusive. Thus, the same tracker features that in one situation are a welcome intervention can in another be viewed in a negative light and violate the sense of self‐determination.

Ultimately, the devices attached to the behavioral insurance products do not exert an especially powerful hold over customers. This became apparent when some participants reported that they had simply stopped engaging with the insurance‐related tracking tools. For others, their use had become habitual. The devices teach people about their daily rhythms; thus, when a person tracks their behavior for a sufficient period, they may no longer need the information that the device has to offer. Some, however, reported that they had stopped tracking because of lackluster experiences with behavioral policies. They were not sure whether their tracking practices really mattered, as they did not receive substantial feedback from the insurance company; what little communication there was felt deeply impersonal. Because they did not receive reminders when they stopped using the devices, some began to think that the tracking features were merely a marketing gimmick without any real effect. Finally, research participants recounted having simply forgotten purchasing a behavioral policy and only remembered it in the focus group. This was true for Hanna, a Company Z customer who said that the behavioral policy “was very easy to start using” but in the end, as she did not investigate it “very carefully” and “did not understand how the collection of activity points worked”, she stopped using the device and “completely forgot” that she even had it. These kinds of experiences were shared in several focus groups: some people had not been engaged to begin with, and if the policies were not attractive or readily comprehensible, people had simply stopped using the tracking features. As the insurance contract does not make tracking activities mandatory—and cannot do so, per EU regulations and Finnish law—there is no way to force people to use them.

## 8 CONTINUING TROUBLE WITH AUTONOMY

The industrial hype concerning digital technologies, including new algorithm‐based behavioral tools, has raised critical debates and promoted ethical precautions and attempts to regulate the rapid developments in this sphere. Thus far, however, it is largely an open question how the new devices actually change people's practices and their relationships with financial products like insurance. In this paper, we have explored how the use of data tracking devices in life insurance policies both widens and narrows customers' scope of action. Our main finding is that when decisions are delegated to algorithmically controlled systems, the notion of autonomy becomes activated in a new manner in the insurance context. In their daily lives, people tend to think little about their traditional insurance policies. As Jeanningros and McFall (2020, p. 12) point out, “one of the most interesting things about insurance is that it’s not interesting”. For the industry, an important promise of algorithmic tools is that they make insurance more engaging by enabling closer communicative relationships between companies and customers (Tanninen et al., 2021). However, as we have shown, the mediation of insurance relations by behavioral tools brings with it a heightened awareness of the (threatened) limits of autonomy. In other words, these new technologies have the unintended consequence of *forcing* people to consider the situational dynamics of autonomy and to engage with questions related to self‐determination. Insurance becomes interesting—yet not as insurance, but as trouble with autonomy.

In the focus group discussions, autonomy was revealed to be a complex concern. On the one hand, individual autonomy was regarded as an entity that can be “had” and “exerted”, offering a criterion for demarcating acceptable control. This juridical form of autonomy reproduces the historically rooted liberal traditions and structures in how the insurance industry and its regulators approach the issue. On the other, our empirical materials make clear that this approach fails to recognize the richness of the trouble that people have with autonomy when they weigh the pros and cons of persuasive technologies. From this perspective, we have analyzed how autonomy is sensed and enacted in relations and collaborations with devices. Instead of taking autonomy as a self‐evident entity, we have demonstrated how it is defined, negotiated, and done in practice. These findings are particularly important for establishing that both industry insiders and social critics tend to have limited views of the notion of autonomy, with the former suggesting that these new tools enable people to become freer to choose how to take care of themselves and the latter merely decrying the controlling elements that are evident in the technology. What both perspectives miss is the ambivalence displayed in real‐world situations where autonomy is constantly experienced and negotiated.

The methodological strength of our study thus lies in rooting the analysis in ongoing ethico‐political deliberation. We demonstrate that rather than a struggle between pure individual autonomy and total submission to the machine, what is at stake is a more contextual zone of contrasting desires and situational negotiations and practices. One can be more or less autonomous in particular situations, not only in relation to other people, institutions, and technologies, but also in an entangled interaction with these other actors—and with their help.

Overall, our analysis shows that if consumers feel that they benefit from tracking features and retain final decisional power over their self‐monitoring practices, they are open to behavioral insurance policies and at times even find them interesting and enjoyable. However, aligning user aims with the goals of the insurance arrangement is difficult to achieve and sustain: tracker qualities that are experienced as helpful at one moment can easily become unwelcome at another. In the latter instances, the upsetting side of control rapidly becomes visible; instead of serving as an external aid that helps people reach goals and enhance self‐determination, the device fails to understand their everyday aims and needs. This causes unpleasant feelings and disturbs the socio‐technical enactment of autonomy.

## 9 CONCLUDING REMARKS

We have called for social scientists to intervene in the current socio‐technical debate by substantiating that shared values are emergent and respond to life's shifting circumstances. This means that the disturbing effects and affects caused by technologies need to be considered when algorithmic systems are evaluated and implemented. Our analysis confirms that autonomy as a notion cannot be solely related to rational sensemaking; instead, emotional responses act as indicators of whether a desirable version of autonomy is being “done” in a particular situation. We have highlighted the role that affective orientations play in determining the limits of acceptable control and suggested that we can use emotions and affects as navigational aids when evaluating the new ways for people to be that emerging technologies promote. In personal reflections, autonomy refers to a sense of being in charge and prepared for whatever comes next. For algorithmic encounters to appear gratifying, people must feel that their self‐determination is not unpleasantly infringed. Losing autonomy temporarily or partially is not an unpleasant experience if it supports an overall appreciation of what lies ahead. One can be told what to do by a self‐tracking device and still feel that one is in command—if one has actively and freely chosen to obey. However, the more consumers are pushed and prodded by algorithmic techniques, including behavioral modification tools, the more they experience trouble with autonomy. Mechanical encounters that do not support the alignment of technologies with users' goals trigger reflections on whether free will is truly free and whether practices and desires are genuinely self‐chosen.

The coexistence of different conceptions of autonomy and the richness of affective discourse in our site of behavior‐based insurance illustrate the need for regulatory and ethical approaches to remain sensitive to different and even contrasting versions of values. In the longer term, rewarding engagements with technologies can only be maintained with alignments that respect notions of personal autonomy. Efforts to safeguard commonly shared values from the negative consequences of algorithmic technologies remain ineffective if they rely on rigid and predetermined understandings of values, disregard their affective dimensions, and provide only sharply demarcated codes of conduct. In line with Rességuier and Rodrigues (2020, p. 3), we see that straightforward ethical principles are not adequate for analyzing and understanding complex issues like autonomy if they do not regard their attachment to intricate relations, emotional landscapes, and socio‐technical systems. Research, ethical considerations, and governance initiatives should all actively create space for considering how values are done in various situated practices and which of those enactments are desirable. This means considering how people actually operate and feel in technologically mediated relations rather than simply promoting regulatory measures based on expected, even ideal, behaviors. The qualities that make us distinctly human—like the ability to reflect on choices and actions and our ambivalent pursuit of self‐determination—should not be bypassed in regulatory debates. Paying attention to moments of alignment and friction with algorithmic systems offers much‐needed guidance for thinking about how to steer ourselves toward more liveable socio‐technical futures where people's boundaries, values, and wills are respected.

## CONFLICT OF INTEREST

The authors declare no potential conflicts of interest with respect to the research, authorship, and/or publication of this article.

## Data Availability

The data that support the findings of this study are available from the corresponding author upon reasonable request.
